# Introductory Lectures

**Published:** 1846-02

**Authors:** 


### INTRODUCTORY LECTURES.

Our notices of these productions must necessarily be brief and superficial, but we are not willing to let any with which the authors favor us pass entirely unnoticed. Many of them abound in valuable thoughts, and are written in a pure and elevated style.

Professor Harrison, of the Ohio Medical College, selected as the theme of his discourse the "Sources, Evils, and Correctives of Professional Discontent"—a subject in which all of our readers necessarily feel a lively interest. It comes quite home to their business and bosoms, and if the lecturer can point to a safe, certain, and pleasant remedy, he will receive the abiding gratitude of the profession. We cannot say that he has discovered a specific, much less a *panacea,* but we take great pleasure in saying that his sentiments are admirable, and although his style is not free from the tincture of which we have sometimes spoken, his lecture will exercise a beneficial influence upon those who listened to it, as it was delivered by its eloquent author, and will fortify and encourage any desponding or querulous member of the healing art who reads it. Whatever may be thought of some of Prof. H.'s theoretical views on medicine, there can be but one opinion respecting his sentiments on moral and religious subjects. They are just and elevated, such as become a teacher in his responsible station.

Professor Linton, of the St. Louis University, delivered the introductory lecture for his colleagues this season, and we have had the pleasure of perusing it. Much of the discourse is taken up with a eulogy on chemistry, which Prof. L. holds should be taught in every large city "at the public expense." We hope the eloquent remarks of the professor will inspire his pupils with a just admiration for this science, which, we suppose we are fully warranted in saying, is generally very much underrated by students and practitioners of medicine. Towards the close of his lecture the writer becomes metaphysical, and at last controversial. His school has been assailed, and he claims the right of defending it, which he does in a right manly spirit. He compliments the "Sisters of Charity" for the self-sacrificing spirit with which they devote themselves to the afflicted, one of the results of which is the erection of Hospitals and Asylums in which medicine is most advantageously taught. If he manifests more warmth of feeling, in some passages, than seems consistent with good taste, let it be remembered that he was speaking in defence of an institution in which his whole soul is enlisted.

Professor Bedford's introductory lecture in the New York University has been for some weeks on our table. It is devoted to the defence of Obstetric Medicine, and an exposition of the responsibilities and duties of the accoucheur. The principles of action are laid down, and then enforced by examples. Some of the illustrations are shocking enough. One woman is mentioned, whose uterus was ruptured by the violence of the operator, the small bowels escaping at the rent, while the body of the fœtus was removed, and the head left in the cavity of the womb! The lecture is rendered graphic and practical by the history of other cases, and, altogether, is a vivid and instructive discourse.

